# VA Research and Operations Uniting to Combat COVID-19 Inequities

**DOI:** 10.1089/heq.2023.0002

**Published:** 2023-05-26

**Authors:** Sarah C. Leder, Justin M. List, Rachel Chandra, Kenneth T. Jones, Ernest Moy

**Affiliations:** Office of Health Equity, Veterans Health Administration, Washington, District of Columbia, USA.

**Keywords:** COVID-19, Veterans, health disparities, minorities, health care

## Abstract

As novel coronavirus 2019 disease (COVID-19) began to spread across the United States in early 2020, health care systems faced extreme demands on resources. As the country's largest single-payer health care system, the U.S. Department of Veterans Affairs (VA) was uniquely positioned to study how the virus impacted different communities and work to improve care provided to all. Early on, a literature review of prior epidemics revealed that occupational exposures and an inability to socially distance could impact some groups more than others. The VA's Office of Health Equity leveraged a general sense of community to create a collaborative research space and a dedicated analytic space to inform pandemic operations. VA researchers and operations staff were able to quickly share information and respond to updates to produce accurate and reliable publications for medical professionals and the general public. Partnerships with VA Medical Centers and Veteran Service Organizations helped to increase communication across the nation and determine the most critical needs. Although COVID-19 was dynamic in nature, VA's intentional examination of social and structural factors was crucial in informing a more equitable approach. Moving forward, these inequities must be intentionally addressed in future pandemic responses.

## Overview

When the novel coronavirus 2019 disease (COVID-19), caused by the severe acute respiratory syndrome coronavirus 2, appeared across the United States in early 2020, many public health and health care systems rapidly experienced unprecedented demands on resources. At the same time, data showed that COVID-19 affected Americans at disproportionate rates; infection rates, morbidity, and mortality all impacted minority and at-risk populations incommensurately.^1–5^ As the largest integrated health care system in the United States, the Department of Veterans Affairs (VA) immediately initiated plans to mitigate COVID-19 inequities that could affect Veterans and the American public.

Established in 2012, the Office of Health Equity (OHE) was one of the offices activated to ensure all Veterans had equitable access to COVID-19 information, testing, and treatment. OHE champions the elimination of health disparities and achieving health equity for all Veterans. The VA Health Equity Action Plan serves as OHE's guiding document for equity implementation and sustainment.^6^ Achieving health equity requires valuing everyone equally with focused and ongoing tailored efforts to address avoidable inequities, historical and contemporary injustices, and the elimination of health and health care disparities.^7^ Since OHE's health equity processes and procedures were already well established, scaling to demand during the COVID-19 pandemic was manageable.

In this article, we discuss how VA leveraged existing resources and designed new approaches to advance health equity by providing equitable COVID-19 care for all Veterans given known marginalizing risk factors that many experience. We describe (1) responses by VA and OHE supporting equity in the design of VA COVID-19 operations; (2) the use of data and health services research to gauge the success of efforts and areas of need in real time; and (3) strengths, areas for improvement, and lessons learned that other health care systems might use for informing future work in ensuring health equity during a pandemic scenario.

## Understanding COVID-19 Inequities

As early as March 2020, anecdotal information suggested COVID-19 was disproportionately affecting people of color and those residing in low-income neighborhoods. Leveraging its electronic health records, VA was able to calculate rates of infection, treatment, and death among Veterans cared for by VA. For example, Rentsch et al. found that in the first 6 months of the pandemic, Black and Hispanic individuals who received care in the VA were more likely to test positive their non-Hispanic White counterparts, but that 30-day mortality did not differ.^8^ Other researchers quickly confirmed these inequities among Veterans.^9–16^ Determined to minimize disparities in COVID-19 among Veterans, VA embarked on a multipronged effort to understand COVID-19 inequities.

The VA Evidence Synthesis Program conducted a rapid literature review of prior epidemics in the United States to provide insight on how COVID-19 might affect minority populations.^17^ Although this report was not focused specifically on Veterans, it provided valuable insight about “potentially modifiable factors”^17^^(p.7)^ that may have played a role in health outcomes and infection rates in previous epidemics. Findings included that minority populations and those of lower socioeconomic status were more likely to be infected and less likely to be vaccinated. Importantly, it suggested that higher infection rates among people of color and those residing in low-income neighborhoods were primarily related to occupational exposures and inability to socially distance.^17^ Applying these findings to the COVID-19 pandemic response suggested that additional outreach to minority Veterans groups, addressing specific concerns, would help ensure that all populations had accessible and accurate information and medical care.

VA asked OHE to establish a dedicated analytic space with complete COVID-19 Veteran data to inform pandemic operations. Staff in OHE set up a health equity COVID-19 database to meet those needs based on case data gathered by the VA National Surveillance Team (NST). The first step was to extract available information on patients who received COVID-19 testing, the results from those tests, and any COVID-19-related laboratory work. The initial NST tables contained the patient's age, gender, death status, and Veteran status. Next, OHE included equity measures, including patient's race/ethnicity, geographic information, service connectedness, period of service, body mass index, and >30 medical and mental health conditions (e.g., hypertension, diabetes, serious mental illness, and other mental health concerns).

Health equity staff used a similar approach for organizing vaccination data and vaccination tables, including the patient age, sex, and race/ethnicity. Staff then set up a system where partner researchers and program offices could request access to the database and tables. A comprehensive health equity COVID-19 data SharePoint site stored codebooks, research articles, and other materials relevant to COVID-19 and health equity. Staff refreshed testing and vaccination tables weekly.

The analytic space was coordinated by Dr. Donna Washington, Director of the OHE/Quality Enhancement Research Initiative National Partnered Evaluation Center, enabled OHE to create a VA-wide equity data community early in the pandemic. It invited researchers to put forth efforts in exploring health inequities and COVID-19, encouraged collaboration, strategic planning, and minimized duplication.

## Sharing Information on COVID-19 Inequities

Early in the pandemic, OHE collaborated with the Assessing Circumstances and Offering Resources for Needs Initiative in Veteran Integrated Services Network (VISN) 1 to create a screener for COVID-19 social risk factors for VA employees to use when interacting with Veterans.^18^ This screener, which was eventually developed into a script that staff assigned to triage incoming phone calls could use during conversations with Veterans who reported symptoms or an exposure, captured information about frequency of leaving home for work and critical activities such as picking up groceries, traveling for medical care, and using public transportation or ridesharing. The hope was to identify Veterans at high risk of social determinants of health insecurities and provide them with appropriate counseling and services.

**FIG. 1.**
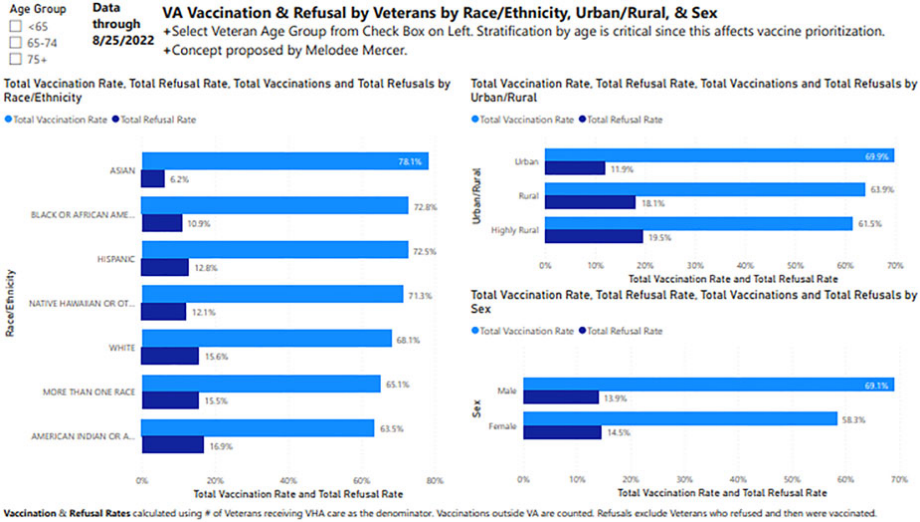
Summary dashboard of VA COVID-19 vaccination/refusal data. VA, Department of Veterans Affairs.

A COVID-19 Equity Dashboard (Fig. 1) visible to all staff allowed VA leaders and providers to identify VISNs and counties with high numbers of community and VA COVID-19 infections. VA patient infections were stratified by race/ethnicity so counties with higher positive test rates among Black, Hispanic, or White Veterans could be targeted for outreach and intervention. When COVID-19 vaccination began in late 2020, COVID-19 vaccination rates by age, sex, race/ethnicity, and rurality were added to the Dashboard. This Dashboard was also used to supplement regular reports to VA leadership and Congress on the state of disparities in COVID-19 care among Veterans receiving care at VA.

In September 2020, OHE hosted virtual listening sessions with Veterans of color with the goal of increasing vaccination rates and determining best practices for disseminating accurate information about COVID-19. These listening sessions shaped VA's tailored messaging to include anticipated Veteran questions and concerns. For example, OHE learned that having professionals with similar demographic backgrounds present information at appointments or meetings with Veterans was crucial for trust-building and addressing vaccine hesitancy. OHE learned that explicitly addressing past injustices such as the U.S. Public Health Tuskegee Syphilis Study and emphasizing VA's role in ensuring all Veterans had the information they needed to make an informed decision on COVID-19 vaccination for themselves and their families was critical when discussing new treatments with minority Veterans.^19^

## Reducing COVID-19 Inequities

VA OHE worked closely with VISNs, VA Medical Centers (VAMCs) across the country, and offices in the VA Central Office to develop tools, strategic plans, and targeted communications and interventions to reach at-risk populations and provide scientific information about COVID-19 and behaviors to mitigate viral spread.^20–22^ Central to OHE's efforts were identifying Veterans who might be at greater risk of serious illness from COVID-19 and working to provide care and messaging to these higher risk populations. OHE accomplished this goal by working in four major areas: a social risks screener, regular updates to VA leadership, operational tracking in the OHE Equity Dashboard, and listening sessions with Veterans.

VA leveraged a general sense of community, fostered at its VAMCs, to prepare staff and Veterans for vaccination by increasing their awareness and knowledge over time. Equity considerations were centered in prioritizing vaccinations for specific groups of Veterans, delivering customized messaging to Veteran groups, and examining needs as the pandemic continued.^10,11,14,23^ Throughout the pandemic, VA approached educational efforts through marketing campaigns, multidimensional outreach programs, and local practices that facilitated ongoing dialogue and a bidirectional exchange of information and perspectives with Veterans and their communities. This comprehensive approach improved disease management, enabled the system to maximize its capabilities, and ensured opportunities to address potential issues of inequity—thus assuring each Veteran of their importance to VA, and VA's willingness to be an active agent for them.

Without considering how social factors intersect, it is impossible to have a comprehensive understanding of what barriers Veterans faced during the pandemic. VA has been working on increasing access to telehealth for Veterans living in rural areas or who have limited broadband connectivity, but “rapid expansion of virtual care was central to the VA national response to the COVID-19 pandemic.”^24^ Yet, Ferguson et al. found that Veterans who lived in urban areas were not experiencing homelessness, or were younger than 45 years were more likely to utilize virtual video care than those who lived in rural areas, were experiencing homelessness, or were over the age of 45.^24^ Although expanding virtual care options increases access, barriers including lack of appropriate devices or software, connectivity issues, and need for technical assistance persist.^24,25^ OHE worked collaboratively with others to address these barriers.

## Outcomes

The VA COVID-19 Response Report, originally released in October 2020, provided a summary of VA's initial response to the COVID-19 pandemic.^20^ Annex A, published in May 2021, highlighted interagency coordination, vaccination planning, clinical operations, and work done to support VA's Fourth Mission, which includes improving U.S. preparedness for national emergencies.^26,27^ Most recently, Annex B was issued in December 2021 and expands on VA's role in mass vaccinations, clinical operations, mental health initiatives, and continued Fourth Mission responses.^28^ Although each of these reports focuses on different time periods, all address aspects of health equity for Veteran populations (race/ethnicity, sex, sexual orientation, age, and rurality).^20,27,28^ Here, we summarize outcomes related to COVID-19 equity.

VA's COVID-19 Equity Dashboard provided VA with specific areas and populations on which to prioritize focus. After the initial COVID-19 surge, testing and mortality disparities related to race/ethnicity were small within VA in contrast to larger differences observed outside VA. VA leadership worked to shrink vaccination rate disparities between Veterans of color and White Veterans. To reach as many different Veteran populations as possible, OHE created educational materials for Veteran populations and their families. Specific population materials included those aimed at Veterans aged 65 and older, pregnant Veterans, Black, Hispanic, and Native American Veterans, Veterans from the recent wars (Operation Enduring Freedom, Operation Iraqi Freedom, and Operation New Dawn), and transplant patients. Ultimately, many groups such as Veterans of color had higher vaccination rates than their White counterparts. However, rural Veterans have persistently had low vaccination rates compared with urban Veterans.

Data also drove the early response to COVID-19 in VA-owned and operated Community Living Centers (CLCs).^29^ Similar to community nursing homes, CLCs were hit hard by COVID-19 and staff “adapted with protocols for symptom monitoring, resident and staff isolation, quarantine, and diagnostic and screen testing.”^29(p.2091)^ CLCs followed Centers for Disease Control and Prevention guidance regarding the prioritization of vaccine delivery and administration to CLC residents and staff.^29^ By virtue of VA's size and national presence, CLCs were better able to navigate the pandemic than some of the smaller community nursing home systems. Personal protective equipment, testing, and vaccinations were all managed on a system level that ensured supply availability to CLCs.^29^

In addition, important information could be shared quickly between CLC staff and VA infection control experts or epidemiologists.^29^ CLCs took significant steps to minimize potential exposure of residents and staff at the beginning of the pandemic, including “restrict[ing] the entrance of nonessential staff to the CLC.”^29(p.2093)^ Combined, these efforts limited the number of positive tests for residents. By December 20, 2020, 50% of CLC residents had received a first vaccination and by January 26, 2021, 82% had begun or completed vaccination.^29^ At this point, positive COVID-19 tests started to significantly decline in VA CLCs and continued to decline for both vaccinated and unvaccinated residents.^29^

In addition to generating information to guide targeted interventions to high-risk groups of Veterans, the VA-wide community of diverse researchers and data analysts published >50 articles that explored how COVID-19 affected various groups of Veterans.^30^ Importantly, a majority of these studies considered how multiple demographic factors (e.g., age, race/ethnicity, sex/gender, location, socioeconomic status, living setting, and specific physical or mental health diagnoses) interacted and impacted Veterans' lives and their experiences during the COVID-19 pandemic.^21^

## Key Takeaways: Addressing Health Equity During COVID-19 Operations

### Challenges

Although several operational components of VA's COVID-19 response were effective in curbing health disparities, there were some significant challenges throughout the course of the pandemic:

- Owing to the inherently dynamic nature of COVID-19 (e.g., shutdowns, reopenings, availability of information, and vaccination), equity needs varied over time as COVID-19 strains developed and disparities across the country changed.
- Misinformation and disinformation: As researchers and policy makers learned more about the virus and announced updated recommendations, the nation, including VA, was challenged due to misinformation and disinformation. Specifically, VA faced challenges in reaching rural veterans and overcoming disinformation about COVID-19 disease and vaccination.

### Strengths

VA's enduring commitment to addressing health equity dimensions in disease prevention and treatment, as well as incorporating equity values into its central mission, supported dynamic and rapid responses to the pandemic. Several effective components of VA's response include the following:

- Leadership understood that evidence-driven decision-making could yield evidence-based tailored interventions. Since VA is the largest integrated health care system in the United States, access to high-quality patient demographic and other data were readily available to provide rates of vaccination for different groups.
- Research and operations staff alike came together to share information and respond quickly to constant updates to produce accurate and reliable publications for both the general public and medical professionals.
- Through partnerships with VAMCs and Veteran Service Organizations, VA increased communication across the country to determine Veterans needs that were most critical. These partnerships also proved key as they served as locations where VA users could access information.
- Use of different modalities of care to reach at-risk populations: VA quickly maximized its technological scope and converted face-to-face appointments to VA Video Connect and telephones to minimize public contact.

### Lessons learned

VA's efforts in managing COVID-19 provide vital lessons for the nation for future epidemics and pandemics. Addressing social and structural disparities must be intentionally incorporated to a pandemic's response strategy at the beginning to ensure it will be more equitable and accessible to more people.

- VA leadership facilitated and coordinated communications to frontline staff, researchers, clinicians, educators, and the VAMC network to adapt as more information was known about COVID-19. Other health care systems may benefit from incorporating flexibility and adaptability to create a strong response to national emergencies.
- VA strongly found that ongoing engagement with target populations and other organizations provided valuable insight for local, state, and national guidance and policy development.

VA serves as a model for its ability to identify and immediately address COVID-19 inequities in its ongoing and timely provision of services throughout the pandemic to both the Veteran population and those cared for through the VA's Fourth Mission. As VA developed strategic plans and communications to handle the pandemic, VA leadership ensured that resources were equitably distributed to minimize disparities.^20,21^ VA also maintained its decade-long practice of incorporating established health equity practices into its clinical treatments, community support programming, and resource allocation processes.

## Abbreviations Used

CLCs: Community Living Centers
COVID-19: novel coronavirus 2019 disease
NST: National Surveillance Team
OHE: Office of Health Equity
VA: Department of Veterans Affairs
VAMCs: VA Medical Centers
VISN: Veteran Integrated Services Network
